# Amyloidosis Localized to the Sinonasal Tract: From the Diagnosis to Management of Disease

**DOI:** 10.7759/cureus.33480

**Published:** 2023-01-07

**Authors:** Sara Gomes, Sara Santos, Monica Silva, Joana Ferreira, Teresinha Ponte

**Affiliations:** 1 Internal Medicine, Centro Hospitalar Barreiro-Montijo, Barreiro, PRT

**Keywords:** tinnitus, nasal congestion, nasopharyngeal amyloidosis, localized amyloidosis, amyloidosis

## Abstract

Amyloidosis is a mainly systemic rare disease and its localized presentation is even less frequent. Systemic forms are often associated with other conditions or entities (such as neoplasms), and a correct etiological study of patients with this disease is essential. A localized presentation has a better prognosis compared to systemic forms, which underlines the importance of a correct diagnosis. Primary localized amyloidosis in the head and neck region is a rare entity. Primary amyloidosis localized to the sinonasal tract is extremely rare. Although uncommon, case reports have been increasing in the face of new endoscopic and imaging techniques, and the report of these cases is crucial for a better characterization of this entity. Symptoms may include epistaxis, nasal obstruction, facial deformity, and vision changes.

A 72-year-old female with a history of frequent nasal congestion with two years’ duration showed no improvement following symptom management. She was referred for nasal endoscopy and biopsy, thereby confirming the diagnosis of amyloidosis. Further diagnostic testing did not show evidence of systemic disease.

This case report was previously presented as a poster presentation at the 19th European Congress of Internal Medicine in March 2021.

## Introduction

Amyloidosis is a rare clinical entity characterized by the deposition of amyloid fibrils and insoluble protein polymers in organs or tissues, leading to cellular injury [1,2]. Diagnosis is based on clinical presentation, symptoms, and histopathology [3]. Amyloidosis can be classified as systemic or localized [4]. Systemic forms are the most frequent, presenting for the most part a worse prognosis than localized forms [2].

Localized presentations are rare, representing about 10% of cases [1,5], and usually have a better prognosis [1]. Most of these are localized to the head and neck region - comprising the larynx, pharynx, eyes, nasal cavity, sinus, and oral cavity [3]. The most frequent site of presentation is in the larynx [1,5,6]. The differential diagnosis (with pathological anatomy and imaging exams) with neoplastic entities is also essential given the different prognoses. According to Tsetsos et al., laryngeal neoplasia is one of the most common forms of head and neck neoplasia, with a survival rate of only 60.6% at five years [7]. In addition to this mortality, patients with laryngeal neoplasia have a high morbidity since they may be subject to a laryngectomy, radiotherapy, or chemotherapy depending on the type of lesion [7].

This clinical case describes a nasopharyngeal presentation, which is extremely rare (3%) [1,4,5], with few cases reported in the literature [2].

## Case presentation

**Figure 1:**
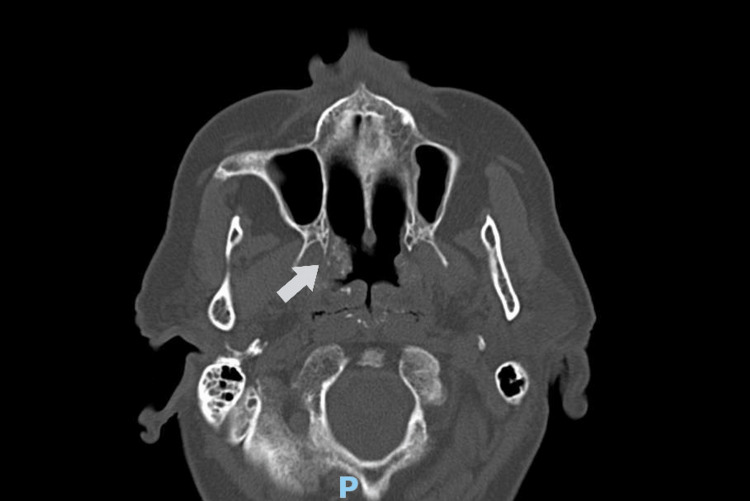
Contrast-enhanced CT scan revealing lymphoid tissue occupying the posterior end of the right nasal cavity CT: computed tomography

The patient was a 72-year-old female with a history of arterial hypertension and dyslipidemia receiving treatment with perindopril, indapamide, amlodipine, and atorvastatin. She had no history of hospital admissions or surgical interventions. Her family doctor referred her to an otorhinolaryngology consultation due to complaints of frequent nasal congestion and ear fullness with two years of evolution. For over one year she underwent symptom management with no improvement. Otoscopic examinations and tympanometry results were unremarkable. Due to the persistence of symptoms, a contrast-enhanced computed tomography (CT) scan of the head and neck was requested, revealing lymphoid tissue occupying the nasal cavity, without muscle tissue, bone, or skull base infiltration (Figure 1). A nasal endoscopy revealed a vegetative lesion at the posterior end of the right nasal cavity extending to the lower contour of the choana. The pharyngeal cavum also presented with lymphoid hyperplasia with small calcifications on the surface. Histopathological examination of the right nasal cavity and pharyngeal region revealed apple-green birefringence on Congo red staining, suggesting amyloidosis.

Subsequently, she was referred to internal medicine for a follow-up. An extensive clinical and laboratory work-up was carried out, including a biopsy of abdominal fat, bone marrow biopsy, serum immunofixation electrophoresis, urine protein electrophoresis, abdominal and renal ultrasound, chest radiograph, coagulation tests, complement factors, electrocardiogram and echocardiogram, liver function tests, renal function tests, serum alkaline phosphatase, and thyroid function tests. No evidence of systemic amyloidosis was found, and the patient was diagnosed as having localized nasopharyngeal amyloidosis.

## Discussion

As previously mentioned, amyloidosis is a rare condition (incidence of 12 cases per million inhabitants per year [3]) whose diagnosis is based on organ involvement and histopathological evidence of amyloid deposits [3]. Localized presentations are rare in the head and neck, with the larynx being the most frequent location (60-61%) [1,2,4] followed by the thyroid gland, the trachea (9%), the orbit (4%), and extremely rare in the nasopharynx (3%) [1,4,6,8,9]. Despite these numbers, there has been an increasing number of reported cases, which may be related to the optimization of imaging and endoscopic techniques [4]. The etiology is unknown and considered idiopathic. A relationship between chronic inflammation and immunological dysregulation has been hypothesized as a possible cause of excess production of precursor proteins with subsequent polymerization into insoluble amyloid fibrils [3,5]. Establishing a demographic profile can be difficult. According to some authors, the youngest patient with the localized nasal form was eight years old, and the oldest was over 80 years old [3,10]. Gender prevalence patterns are also not defined [3], despite some authors referring to a higher rate in females [5,8]. Concerning the symptoms, as mentioned, they are non-specific, with the main presentations being nasal congestion, changes in hearing acuity, otitis media, eustachian tube dysfunction, chronic rhinosinusitis, and epistaxis [4,2,5].

Systemic involvement has to be excluded given the marked differences in morbidity and mortality between these two forms [8]. In its localized form, malignant tumors need to be considered in the differential diagnosis, such as nasopharyngeal carcinoma or lymphoma in the case of nasopharyngeal amyloidosis, and the calcifications observed radiologically increased this diagnostic suspicion. In addition, amyloidosis can be associated with malignancy [4], which further emphasizes the importance of a complete diagnostic work-up and a multidisciplinary approach (in this case with Internal Medicine and Otorhinolaryngology). A biopsy is the only confirmatory method [3], and the Congo red stain technique helped to characterize deposits as amyloid deposits - a technique initially used by Benhoff in 1922 [4]. Abdominal fat biopsy, performed in this case, is as sensitive as rectal biopsy (75-90%) [5,6], having a specificity of 92-100% [8], and being one of the essential tools for diagnosis.

This localized presentation is usually a slowly progressive disease with a benign course [1]. Therapy often involves radiation or surgery and is not usually associated with morbidity and mortality (cardiac and renal) of systemic forms. Surgical therapy requires an assessment of the risks and benefits for the patient since the excision of lesions can contribute to morbidity, impairing the normal physiological functions of the patient. A conservative approach is often taken given the slow development of the disease [4]. The recommended surgical approach is often conservative and not radical, given the high risk of recurrence [2], which according to some authors is 50% [5].

## Conclusions

Although progression to systemic disease is rare, it can occur, which further highlights the importance of a complete diagnostic assessment given the worse prognosis of systemic forms (as was done in this case). Given the possible existence of small lesions not evident through imaging, it is also essential to keep these patients under careful monitoring. The patient presented maintains this follow-up in the Internal Medicine Consultation.
